# Cryo-Technologies for Ex Situ Conservation of *Rosa* Germplasm

**DOI:** 10.3390/plants11081095

**Published:** 2022-04-18

**Authors:** Adela Halmagyi, Sergiu Vălimăreanu, Gabriela Șovărel, Ana Coste

**Affiliations:** 1 NIRDBS, Institute of Biological Research Cluj-Napoca, Republicii 48, 400015 Cluj-Napoca, Romania; adela.halmagyi@icbcluj.ro (A.H.); sergiu.valimareanu@yahoo.co.uk (S.V.); 2 Department of Plant Molecular Biology, Faculty of Biology and Medicine, University of Lausanne, CH-1015 Lausanne, Switzerland; 3 Research and Development Institute for Vegetable and Flower Growing, Calea București 22, 077185 Vidra, Romania; gabriela_sovarel@yahoo.com

**Keywords:** droplet-vitrification, encapsulation-dehydration, genetic resources, long-term storage

## Abstract

In this study, we compare two rapid cryopreservation (−196 °C) procedures, droplet-vitrification and encapsulation-dehydration for rose (*Rosa* × *hybrida* L., cultivars ‘Ioana’, ‘Mariana’, ‘Vulcan’). Significant factors for cryopreservation, such as sucrose concentration during osmoprotection, treatment duration with plant vitrification solution 2 (PVS2) in droplet-vitrification, duration of air desiccation and moisture content of alginate beads in encapsulation-dehydration, were investigated. In addition, the morphogenetic response to in vitro culture and to liquid nitrogen storage and the content in photosynthetic pigments have been assessed. The in vitro cultures were initiated from plant material originating from field collection. The highest regeneration frequencies were obtained for cv. ‘Vulcan’ in both of the cryopreservation procedures tested, 72% in droplet-vitrification and 65% following encapsulation-dehydration. The morphogenetic response (multiplication index and height of shoots) to liquid nitrogen storage was direct multiple shoot formation per initial shoot tip for all genotypes. The content in chlorophyll *a* and *b* was statistically comparable in plant material resulting from cryopreserved and non-cryopreserved shoot tips in all cultivars. The findings expand the information on *Rosa*‘s response to in vitro culture conditions and cryopreservation, providing protocols with a high regeneration capacity for the storage of genotypes with high ornamental value.

## 1. Introduction

Roses are among the most commonly cultivated ornamental plants worldwide [1]. The genus *Rosa* (L.) (approximately 200 species) is widely distributed throughout temperate and sub-tropical habitats [2]. Modern cultivars are mostly interspecific hybrids, which are propagated mainly by cuttings, layering or grafting [3]. In vitro propagation of roses plays an essential role in the rapid multiplication of cultivars with desirable characteristics [4,5]. Great progress has been made in the development of various biotechnological approaches for the propagation and conservation of ornamental plants [6,7,8]. Micropropagation protocols for the *Rosa* species and cultivars have been developed using shoot tips [9], axillary buds [10,11] and somatic embryos [12].

The availability of storage methods for the conservation of genotypes with valuable characteristics represents a requisite for the global floriculture market growth due to the expansion of electronic commerce in recent years [13].

Conventional conservation methods for woody perennials are living gene banks represented by clonal collections of agricultural, horticultural or forest species, which enables the identification of cultivars with traits of interest [14]. Besides the susceptibility to environmental stress factors, diseases and insects [15]. A major disadvantage of such collections is the limitation in terms of genetic diversity [16]. The development of cryopreservation (−196 °C) procedures, which started some decades ago [17,18], represents a security backup for clonal collections [19]. Cryopreservation has been considered to be a good option for the long-term storage of vegetatively propagated woody species, including *Vaccinium* [20], *Rubus* [21], *Ribes* [22], buxus [23], *Malus* [24], *Vitis* [25]. Successful regeneration of *Rosa* species and cultivars following cryostorage has been reported after droplet-vitrification [26,27,28,29,30], vitrification [31] and encapsulation-dehydration [32,33,34,35]. Although some ornamental crops (cut flowers or potted plants) are intensive crops, which are produced throughout the year, the use of conserved plant material upon market requirements leads to saving resources used [36].

Considering the above issues, the specific objectives of this study were: (a) to evaluate the in vitro morphogenetic response according to the explant type, (b) to compare two cryopreservation approaches, droplet-vitrification (DV) and encapsulation-dehydration (ED), analyzing procedure-related parameters with their influence on shoot regeneration following cryostorage, (c) to determine the morphogenetic response following cryopreservation by DV and ED, and (d) to assess the content in photosynthetic pigments following cryostorage.

## 2. Results

### 2.1. In Vitro Culture Initiation and Micropropagation

**Figure 1:**
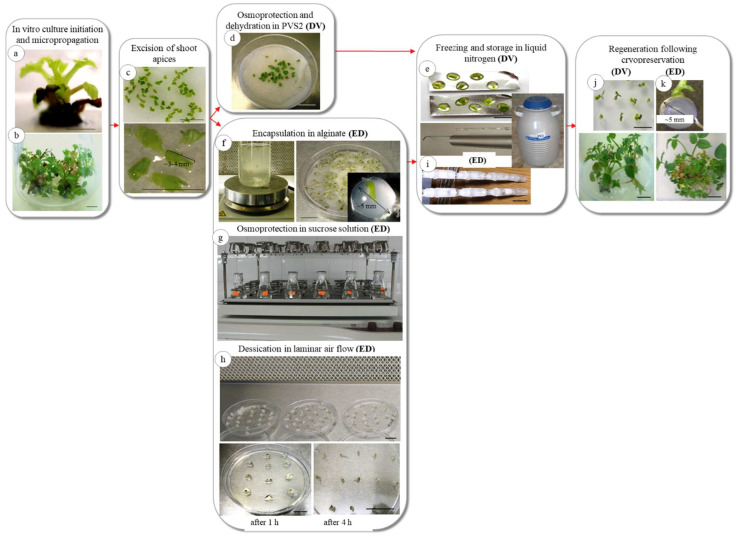
The main steps of cryopreservation procedures (droplet-vitrification: DV, and encapsulation dehydration: ED) are applied for *Rosa* genotypes. (**a**) Shoot regeneration from nodal explants for in vitro culture initiation; (**b**) in vitro shoot multiplication; (**c**) shoot apices excised in sterile conditions; (**d**) osmoprotection in sucrose solution followed by dehydration in PVS2 solution; (**e**) shoot apices in drops of PVS2 solution on aluminum foil strips prepared for freezing; (**f**) polymerization of alginate beads, including shoot apices on a shaker (left image) and alginate beads on filter paper (right image); (**g**) osmoprotection in sucrose solution on a rotary shaker; (**h**) alginate beads in laminar air flow for desiccation; (**i**) cryotubes with samples prepared for immersion in liquid nitrogen; (**j**) shoot regeneration following cryostorage by DV; (**k**) shoot regeneration following cryostorage by ED. Bars = 1 cm.

In the case of in vitro culture initiation, the first signs of shoot growth from apical and nodal segments (approximately 2 cm in length) were observed after three weeks of the explants being in culture (Figure 1a). The morphogenetic pattern and the in vitro regeneration capacity showed no differences based on the explant type (Table 1). Both apical and nodal explants in all cultivars showed a multiplication index five weeks after subculture (Figure 1b). The highest number of shoots per explant (6.8) and the maximum height of shoots (6.1 cm) were recorded for apical explants of cv. ‘Vulcan’ (Table 1). Shoots derived from both explant types were vigorous, but those resulting from the apical explants showed a faster growth. A strong positive correlation was evidenced between the shoot regeneration percentages and the number of shoots per explant (Pearson’s correlation coefficient was 0.99 for apical explants and 0.88 for nodal explants) for the three genotypes. The shoot regeneration from apical and nodal explants was significantly different only for cv. ‘Ioana’, whereas no significant differences were noted for the number and the height of shoots. Neither spontaneous root formation nor callus growth was observed.

### 2.2. Cryostorage

#### 2.2.1. Regeneration following Droplet-Vitrification (DV)

**Figure 2:**
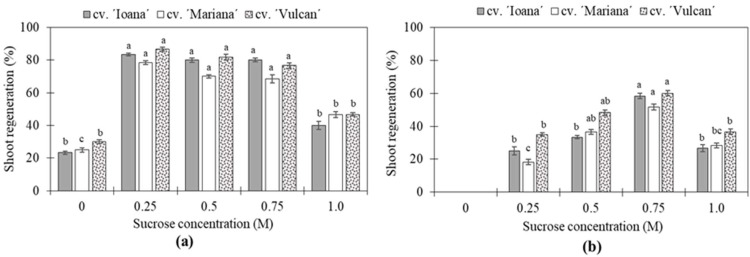
Effects of sucrose concentration on shoot regeneration of (**a**) non-cryopreserved and (**b**) cryopreserved shoot tips. Osmotic dehydration was performed in a liquid MS medium containing sucrose (0.25, 0.5, 0.75, 1.0 M) for 24 h, followed by 20 min PVS2 treatment. Vertical bars represent SD; Different letters indicate significant differences between treatments within the same cultivar (*p* ≤ 0.05).

The sucrose concentration was critical for high regeneration frequencies even for non-cryopreserved shoot tips (Figure 2a), and the importance of concentration was more obvious after cryopreservation (Figure 2b). For all cultivars, significant differences for non-cryopreserved (−LMLN) shoot tips were noted for treatments with zero and 1.0 M sucrose (Figure 2a). The highest regeneration frequencies after cryopreservation (58% for ‘Ioana’, 52% for ‘Mariana’ and 60% for ‘Vulcan’) were obtained after 24 h osmoprotection in 0.75 M sucrose at 23 ± 1 °C in light conditions (Figure 2b). For cryopreserved shoot tips (+LN), the differences were significant within the same cultivar for the tested sucrose concentrations (Figure 2b). It was evident that PVS2 without previous osmotic dehydration in sucrose exerted harmful effects on shoot regeneration, obtaining regeneration rates between 33% (‘Mariana’) and 38% (‘Vulcan’) for non-cryopreserved (−LN) shoot tips (Figure 2a), whereas no regeneration was noted for cryopreserved (+LN) shoot tips in the absence of sucrose (Figure 2b).

Cryoprotectant exposure time was critical for the regeneration of shoot tips following liquid nitrogen storage. The duration of plant vitrification solution 2 dehydration, which followed osmoprotection in 0.75 M sucrose for 24 h, considerably affected the regeneration of cryopreserved shoot tips. Significant differences in shoot regeneration were noted for the various dehydration times, whereas the highest regeneration frequencies following cryopreservation ranged between 58% (‘Mariana’) and 72% (‘Vulcan’) for 30 min of PVS2 treatment (Table 2, Figure 1i). The regression analysis showed a significant strong, positive linear correlation between regeneration frequencies of non-cryopreserved explants and the dehydration duration (r^2^ = 0.93 cv. ‘Ioana’, r^2^ = 0.87 cv. ‘Mariana’, r^2^ = 0.92 cv. ‘Vulcan’). Instead, the correlation was moderately positive between regeneration frequencies of cryopreserved explants and the dehydration duration (r^2^ = 0.41 cv. ‘Ioana’, r^2^ = 0.54 cv. ‘Mariana’, r^2^ = 0.57 cv. ‘Vulcan’). For all cultivars, the dehydration time for high regeneration frequencies after cryopreservation was 30 min. For cryopreserved shoot tips (+LN), no regeneration was found without PVS2 dehydration regardless of genotype (Table 2). Lower regeneration percentages (8% in cv. ‘Ioana’) or no regeneration (‘Mariana’) after cryopreservation were obtained for the 10 min dehydration duration (Table 2). Similarly, an increased dehydration duration (40 min) led to low regeneration rates after cryopreservation (up to 21% in cv. ‘Vulcan’) (Table 2).

#### 2.2.2. Regeneration following Encapsulation-Dehydration (ED)

**Figure 3:**
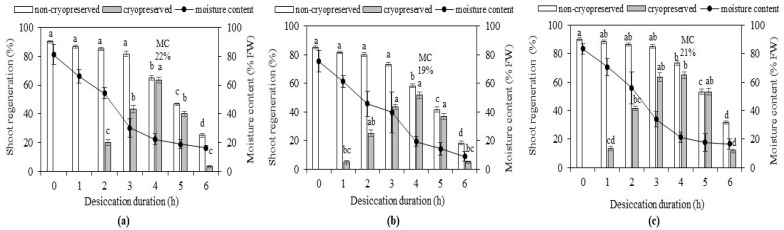
Shoot regeneration from alginate-coated non-cryopreserved (−LN) and cryopreserved (+LN) shoot tips according to the bead moisture content (MC) and the desiccation duration: (**a**) cv. ‘Ioana’, (**b**) cv. ‘Mariana’ and (**c**) cv. ‘Vulcan’. Osmoprotection was made in 0.75 M sucrose solution for 24 h at 23 ± 1 °C during a 16 h light photoperiod. The indicated MC values represent percentages leading to the highest regeneration rates for each cultivar. Vertical bars represent standard deviation. Different letters indicate significant differences (*p* ≤ 0.05).

In the ED procedure, the moisture content of the beads was assessed at hourly intervals (0 to 6 h) for all cultivars. The initial water content of the beads ranged between 75% (cv. ‘Mariana’) and 83% (cv. ‘Vulcan’) and decreased gradually with the increase of the desiccation time to a minimum of 10% (cv. ‘Mariana’) after six hours of air desiccation (Figure 3a–c). Shoot regeneration from cryopreserved (+LN) alginate-coated shoot tips was closely related to the moisture content of the beads, increasing along with decreased bead moisture content up to 4 h desiccation. The moisture content that led to the highest regeneration rate (65% for cv. ‘Vulcan’) after liquid nitrogen storage was 21% after 4 h desiccation (Figure 3c). Significant differences were observed for cryopreserved shoot tips within the same cultivar for different desiccation times (Figure 1j and Figure 3a–c).

The morphogenetic response to LN storage was direct multiple shoot formation for all cultivars (Table 3). No callus development was observed. We observed that shoots resulting from DV showed faster growth than shoots resulting from ED. For example, 30 days after rewarming the shoot tips from DV, they had a length of approximately 0.5–1 cm more than shoots after ED (data not shown). As in the case of micropropagated plants, no spontaneous root formation was observed for shoots regenerated after cryostorage in none of the tested procedures. Significant differences were noted for the number of shoots regenerated from cryopreserved (by both procedures DV and ED) shoot tips for cvs. ‘Ioana’ and ‘Vulcan’, whereas no significant differences were obtained for the height of shoots (Table 3). The number of shoots/explant was between 4.8 (cv. ‘Ioana’) after ED and 6.5 (cv. ‘Vulcan’) after DV (Table 3).

### 2.3. Photosynthetic Pigment Content

The content of chlorophyll *a* and *b* showed no significant differences in plant material resulting from cryopreservation (DV and ED) compared to the chlorophyll content in leaves from non-cryopreserved shoot tips for all cultivars. Carotenoids showed significant differences for cultivars ‘Ioana’ and ‘Vulcan’ in both cryopreservation procedures (Table 4).

## 3. Discussion

The availability of storage methods for the conservation of genotypes with valuable characteristics represents a requisite for intensive breeding programs, which require access to extensive genetic resources [4,37]. The establishment of long-term conservation methods becomes even more important in the frame of the global COVID-19 pandemic [38]. Germplasm conservation strategies are mainly oriented towards food crops due to their importance for food security. Thereby, worldwide, in field gene banks are preserved crops, such as potato, banana, apple, citrus and coffee [38]. No concerted efforts have been made to the conserve genetic resources of ornamental species [39]. The gap between germplasm conservation strategies for food plant species and ornamentals cannot be overlooked.

For roses, there are many reports on direct in vitro shoot proliferation [5,40], whereas multiple shoot formations from different explant types is a common aspect [41,42]. Regarding shoot multiplication, various *Rosa* species and cultivars showed different proliferation frequencies on various culture media. For example, in *R. damascene,* the number of shoots/explant was five in a medium with 4 mg L^−1^ *N*^6^-benzyladenine [43], two shoots/explant in a medium with 0.1 mg L^−1^ gibberellic acid [44] and nine shoots/explant were obtained for rose cv. First Red in a medium with 4 mg L^−1^ benzylaminopurine and 3 mg L^−1^ gibberellic acid [45]. Kapchina-Toteva et al. [46] mentioned that an exogenous cytokinin added to the medium reduces apical dominance, inducing axillary shoot development in roses. In our *Rosa* cultivars, the culture medium with 1.5 mg L^−1^ *N*^6^-benzyladenine led to a shoot proliferation of 6.8 shoots/explant for cv. ‘Vulcan’, 5.4 shoots/explant for cv. ‘Ioana’, and 5.6 shoots/explant for cv. ‘Mariana’ (Table 1).

Although a wide range of cryogenic procedures has been developed for woody species, research that provides optimized protocols is still needed. Sometimes, minor modifications in the pre and post-recovery steps might lead to improvement in the regeneration rates and increase the applicability of cryopreservation for the long-term conservation of the species [38]. The various approaches used for the cryopreservation of ornamental species, as well as the type of plant material used and the achieved survival rates, have been extensively reviewed [47]. Our findings showed that the exposure of shoot tips to a certain sucrose concentration and vitrification time was critical to ensure regeneration, as previously shown in the cryopreservation of other *Rosa* species [28,29,48]. The osmoprotective effect of sucrose may be due to the fact that sugars probably penetrate the cell membrane [49]; as they are the smallest carbohydrates [50]. At the same time, sucrose assures protection for cells against the toxicity of vitrification solution components [51]. The cytotoxicity of cryoprotectants constitutes a challenge in developing cryopreservation procedures, particularly in vitrification-based protocols where high concentrations are necessary to achieve the vitreous state [52]. Due to the heterogeneity of the cells in a tissue, survival and especially regrowth of woody species following cryopreservation could be a challenging issue [20,21]. The shoot tip size was an important factor in regrowth following cryopreservation. For example, applying DV protocols using small shoot tips (1–2 mm in length) resulted in 66% regrowth following cryopreservation for blueberry [20], 43% regrowth for *Vitis* [25] and 40% regrowth for *Rosa* [28], while using large shoot tips (3–4 mm in length) resulted in 75% recovery following cryopreservation for blackcurrant [22], and no regeneration for *Rosa* shoot tips, although 18% of them survived [48]. Compared to our previous results obtained with DV on other rose cultivars using shoot tips of the same size (3–4 mm) or smaller (1–2 mm) [26,27], we obtained higher regeneration percentages (72% cv. ‘Vulcan’) using 0.75 M sucrose and reducing the osmoprotection time to 24 h (instead of 48 h) (Table 2); although there were also other parameters involved, such as the PVS2 dehydration duration. Studies have shown that the regrowth percentages after applying DV in *Rosa* have varied results. For example, in wild roses, the regrowth was 40% [28], in cv. ‘Gold Medal’ 55% [31], and in *Rosa chinensis* 86% [35]. Methods involving alginate encapsulation followed by desiccation have been used in many cryopreservation strategies for a wide variety of plant genetic resources [53] since their first development for potato and pear shoot tips [54,55]. This procedure was applied for various ornamental herbaceous or woody species, such as chrysanthemum [56,57], *Ajania* [58] and buxus [23]. The moisture content of alginate beads, which conducted the highest regeneration percentages, was 22% (63% regeneration for cv. ‘Ioana’), 19% (51% regeneration for cv. ‘Mariana’) and 21% (65% regeneration for cv. ‘Vulcan’) (Figure 3). This percentage of alginate beads’ moisture content is within the limits reported for other woody species. For example, the optimum percentage of moisture content for citrus ranged between 20% and 25% [59], 24% for apple [60] and 38% for *Rosa chinensis* [35]. After applying ED for *R. multiflora*, 25% regrowth was achieved with a moisture content of alginate beads of 15–20% [32]. In the DV procedure, the advantages are a rapid procedure and faster recovery following storage, whereas the main drawback is the toxicity of the vitrification solution. The advantage of the ED technique might be an easier way to implement it when dealing with a large number of explants, while the disadvantage could be a longer recovery period after cryostorage.

The content in pigments in plant tissue exposed to chilling stress showed either no alterations at 15 °C [61] or a decrease in chlorophyll accumulation at temperatures of 18 °C and 12 °C [62]. Likewise, a significant decline in the chlorophyll content was determined in leaves from cryopreservation-recovered plants [57]. In *Rosa* cultivars, the content of chlorophyll *a* and chlorophyll *b* was not significantly different in plant material resulting from non-cryopreserved shoot tips, whereas the amount of carotenoids was significantly different in cv. ‘Ioana’ and cv. ‘Vulcan’ (Table 4). A decrease in the content of green pigments, particularly in chlorophyll *b*, and an increase in carotenoids was reported in *Hypericum* plants regenerated after cryopreservation [63]. Similarly, Zevallos et al. [64] showed decreased content of chlorophylls in plants developed from cryopreserved seeds. Contrary to the above-mentioned results, the chlorophyll content in leaves of *Lupinus* plants recovered from cryopreservation was similar to plants raised from non-cryopreserved plant material [65]. Villalobos et al. [66] mentioned that the chlorophyll content was statistically comparable in sorghum plants recovered from cryopreservation and control plants. The contrasting results may be due to various factors, such as growth conditions or different time duration after cryostorage were selected for pigment content determination. It could be assumed that differences in pigment contents observed shortly after cryopreservation will not be detected in plants after a longer period of in vitro culture.

The *Rosa* cultivars created at the Research and Development Institute for Vegetable and Flower Growing Vidra, Romania, are well adapted to local agro-climatic conditions. The main horticultural characteristics (detailed in Table S1) are: plant growth type, flower type, flower color group, flower diameter and petal number of colors [67]. The height of plants is between 80 and 91 cm. Cultivars ‘Ioana’ and ‘Mariana’ have a medium number of flowers, while cv. ‘Vulcan’ has a very high number of flowers. The floral bud is ovoid (cv. ‘Ioana’ and cv. ‘Mariana’) or globose (cv. ‘Vulcan’), and the flowering period is 104 days for cv. ‘Ioana’, 96 days for cv. ‘Mariana’ and 87 days for cv. ‘Vulcan’. 

The highest regeneration percentages following cryopreservation by DV (65% cv. ‘Ioana’, 58% cv. ‘Mariana’, 72% cv. ‘Vulcan’) were obtained after osmoprotection in 0.75 M sucrose for 24 h and 30 min dehydration in PVS2. In ED, osmoprotection in 0.75 M sucrose for 24 h followed by 4 h desiccation led to the highest regeneration percentages, 63% (22% MC) cv. ‘Ioana’, 52% (19% MC) cv. ‘Mariana’ and 65% (21% MC) cv. ‘Vulcan’. In both procedures, regeneration took place at 23 ± 1 °C during a 16 h light photoperiod.

## 4. Materials and Methods

### 4.1. Plant Material, Culture Conditions, Micropropagation

For micropropagation and cryopreservation studies, three *Rosa* × *hybrida* (L.) genotypes have been selected based on their horticultural characteristics (Table S1). The cultivars ‘Ioana’, ‘Mariana’ (both homologated in 2002) and ‘Vulcan’ (homologated in 2000) belong to the *Thea hybrida* group of garden roses and were created at the Research and Development Institute for Vegetable and Flower Growing Vidra, Romania. The morphological characters were determined according to the UPOV guidelines [67]. In vitro culture initiation was made using stems collected from the field. For surface sterilization, the leaves and spines were removed from stems, which were cut into segments (15–20 cm in length), then washed for one hour in tap water, immersed in a 75% sodium hypochlorite (5% active chlorine) solution for 20 min and rinsed three times with sterile distilled water. The explants shaped in apical and nodal segments (approximately 2 cm in length) were transferred to glass containers (3 cm diameter/12 cm height sealed with plastic foil, one explant per jar) containing a previously autoclaved (for 20 min at 121 °C) Murashige and Skoog [68] (MS) medium with 20 g L^−1^ sucrose and 7 g L^−1^ agar without growth regulators (the pH was adjusted to 5.7 before autoclaving) for initiation and shoot elongation (Figure 1a). The cultures were grown at 23 ± 1 °C during a 16 h light photoperiod with a light intensity of 40 mmol m^−2^ s^−1^ photosynthetic active radiation provided by cool white fluorescent tubes. After 40 days, from the newly formed shoots, explants were transferred to 100 mL Erlenmeyer flasks (2 explants per container) on MS medium supplemented with 1.5 mg L^−1^ *N*^6^-benzyladenine, 0.5 mg L^−1^ indole-3-acetic acid, 20 g L^−1^ sucrose and 7 g L^−1^ agar (pH 5.7) for multiplication (in 100 mL Erlenmeyer flasks two explants per vessel) (Figure 1b). The plant growth regulators and their concentrations in the micropropagation medium were selected based on previous results (unpublished data). Subcultures were performed every 5 weeks. 

### 4.2. Cryopreservation Procedures

For cryostorage studies, the explants were excised from *Rosa* plants micropropagated (as mentioned above) for 2 years with subcultures every 5 weeks. Individual shoot tips (apical dome with 2–4 leaf primordia, approximately 3–4 mm in length) were dissected from 3-week-old in vitro plants under a stereomicroscope in sterile conditions. Two rapid cooling approaches have been compared, droplet-vitrification (DV) and encapsulation-dehydration (ED).

#### 4.2.1. Droplet-Vitrification

For this procedure, a protocol described for other *Rosa* genotypes [26] was applied. In this study, only large (3–4 mm in length), apical shoot tips were placed in a liquid MS medium containing sucrose (0.25, 0.5, 0.75, 1.0 M) (pH 5.7) for 24 h at 23 ± 1 °C in light conditions (Figure 1c). After incubation, the shoot tips were placed in the previously filter-sterilized plant vitrification solution 2 (PVS2) [69] at 23 ± 1 °C for 0, 10, 15, 20, 25, 30, 35 and 40 min (Figure 1d). For experiments regarding the effects of sucrose concentrations on shoot regeneration of non-cryopreserved (−LN) and cryopreserved (+LN) shoot tips, the PVS2 treatment was 20 min based on previously obtained results [24]. For cooling the explants were individually placed in a drop (6 μL) of PVS2 on previously sterilized (4 h at 180 °C) aluminum foil strips (0.5/2 cm) and were transferred to 2 ml cryovials (2 foils per cryovial with 5 shoot tips per foil) (Figure 1e). The cryovials were immersed in liquid nitrogen (LN) contained in a 25-L Dewar flask, where the samples remained for 24 h. The rewarming of samples was performed by transfer of the aluminum strips containing the shoot tips to a liquid MS culture medium with 20 g L^−1^ sucrose (without growth regulators) (pH 5.7) at 23 ± 1 °C. By gentle shaking of the aluminum strips in the liquid medium, the drops melted instantly, and the shoot tips were quickly removed. For shoot regeneration, the controls and rewarmed shoot tips were transferred to Petri dishes (5 cm in diameter) on the above-mentioned medium with 6 g L^−1^ agar under the above-described growth conditions.

#### 4.2.2. Encapsulation-Dehydration

An encapsulation-dehydration protocol previously described [60] was applied. For encapsulation, the shoot tips were plunged into a solution of 3% (*w*/*v*) sodium alginate in Ca^2+^-free MS liquid medium. Drops of alginate solution with explants were sucked into a micropipette with sterile plastic tips and dropped into MS liquid medium supplemented with 100 mM calcium chloride (CaCl_2_ × 2H_2_O) under continuous stirring (Figure 1f). After 25 min of polymerization, the beads (approximately 0.4–0.5 cm in diameter) were rinsed three times with sterile distilled water to remove traces of calcium chloride. The alginate beads were harvested by filtration. All operations were performed under sterile conditions. Encapsulated shoot tips (Figure 1f) were incubated in a liquid MS medium containing 0.75 M sucrose (pH 5.7) for 24 h on a rotary shaker (98 rpm) at 23 ± 1 °C. This sucrose concentration was selected due to the good results obtained in the DV procedure. In the ED approach, osmoprotection was carried out after the encapsulation of shoot tips. Encapsulated shoot tips were then desiccated in laminar air flow for up to 6 h (Figure 1g). During desiccation, the environmental conditions in the room were monitored for temperature (23 ± 1 °C) and relative humidity (39%). At 1-h intervals, desiccated beads were placed in 2-ml cryovials (5 beads/cryovial) and immersed in liquid nitrogen (LN) (Figure 1h). After 24 h storage, rewarming was performed by immersion of tightly closed cryovials in a water bath at 38 °C for 2 min. For shoot regeneration, the encapsulated shoot tips were transferred to the same medium and growth conditions as mentioned for the DV procedure. The moisture content (MC) of encapsulated shoot tips was assessed for all cultivars. For dry weight (DW) determination, 10 beads per treatment were weighed and dried at 60 °C until constant weight was attained. The percentages of moisture content were related to the entire bead and were expressed on a fresh weight basis.

### 4.3. Assessment of Photosynthetic Pigments

The content in chlorophyll (*a, b*) and carotenoids was assessed in plant material resulting from non-cryopreserved (−LN) and cryopreserved (+LN) shoot tips three months after rewarming. For each genotype, three randomly selected individual shoots were used, and four leaves from the central part of each shoot were analyzed. The extraction was performed in N, N-Dimethylformamide according to Wellburn [70]. The pigment quantification was spectrophotometrically (*Metertech* SP-8001 UV/Visible *Spectrophotometer)* performed at 664 (chlorophyll *a*), 647 (chlorophyll *b*) and 480 nm (carotenoids) wavelengths [70], and was expressed in mg g^−1^ fresh weight (FW) according to the formulas [70]:

### 4.4. Data Collection and Statistical Analysis

The in vitro shoot induction rate was assessed 30 days after the transfer to the culture medium (*n* = 20 with 3 replicates), according to the formula:

Shoot induction rate (%) = mean number of explants showing growth (˃0.5 cm in length)/total number of explants × 100;

The multiplication index was considered as the mean number of newly formed shoots (˃1.5 cm in length) per individual explant. This parameter was determined 60 days after rewarming (*n* = 20 with 3 replicates). The height of shoots was assessed (5 weeks after rewarming) by removing shoots from in vitro culture and measurement (in cm) from the base of the shoot to the last bud.

Each cryopreservation-related treatment for both procedures was performed using three replicates, each of 10 explants. For the evaluation of regrowth six weeks after rewarming, only shoot regeneration was considered and was defined as the development of shoots with leaf emergence (>1.5 cm in length) from the original explant. Brown shoot apices were considered dead. Data regarding shoot regeneration was expressed as mean percentages according to the formula:

Shoot regeneration rate (%) = mean number of explants showing regrowth/total number of explants × 100;

Dehydration controls (−LN) for both procedures refer to replicates carried out under the same conditions as cryopreservation but without immersion in LN. Hence, osmoprotected (0.25, 0.5, 0.75, 1.0 M for DV and 0.75 M for ED) and dehydrated in PVS2 (for DV) or desiccated in laminar air flow (for ED) explants were used as controls. 

The morphogenetic response of shoot tips following DV and ED expressed as multiplication index and height of shoots was assessed as mentioned above, 60 days after rewarming. The Pearson’s correlation coefficient was determined between the shoot regeneration and the number of shoots per explant for apical and nodal explants independently using the Excel spreadsheet software (v16.0 Microsoft). A correlation coefficient of −1 represents a perfect negative correlation, 0 means no correlation, and +1 is a perfect positive correlation. The regression analysis based on the value of the coefficient of determination (r^2^) was conducted between the PVS2 dehydration duration and the regeneration frequencies for non-cryopreserved and cryopreserved shoot tips using the Excel spreadsheet software (v16.0 Microsoft). The statistical significance of data was determined by one-way analysis of variance (ANOVA) followed by Tukey’s honestly significant difference (HSD) test (PB ≤ 0.05) using SPSS program ver. 17.0 (SPSS Inc., Chicago, IL, USA).

## 5. Conclusions

Shoot tips showed high regeneration following cryostorage by DV and ED regardless of the genotype. No significant cryopreservation and genotype interaction was found for the multiplication index and height of shoots during shoot regeneration following cryostorage. It can be concluded that both procedures applied are efficient and valuable methods for the cryopreservation of *Rosa* germplasm, contributing to the ex situ conservation of ornamental plant germplasms. We aim to implement these cryopreservation approaches, including ex vitro acclimatization, to other ornamental plant species, especially economically valuable genotypes. However, further research should focus on genetic and epigenetic stability studies fundamental for enhanced understanding.

## Figures and Tables

**Table 1:** The morphogenetic response according to explant type.

*Rosa* Genotypes	Explants	Response of Explants		
		Shoot Regeneration (% ± SD) *	Multiplication Index (nr. ± SD)	Height of Shoots (cm ± SD)
‘Ioana’	apical	76 ± 2.5 ^a^	5.4 ± 2.0 ^a^	5.6 ± 1.2 ^a^
	nodal	69 ± 1.6 ^c^	4.2 ± 1.9 ^a^	4.4 ± 1.1 ^a^
‘Mariana’	apical	85 ± 1.5 ^a^	5.6 ± 1.1 ^a^	5.4 ± 1.5 ^a^
	nodal	73 ± 2.5 ^a,b^	4.6 ± 1.9 ^a^	3.8 ± 1.0 ^a^
‘Vulcan’	apical	90 ± 2.3 ^a^	6.8 ± 1.3 ^a^	6.1 ± 2.0 ^a^
	nodal	85 ± 1.8 ^a^	5.6 ± 1.9 ^a^	4.9 ± 1.7 ^a^

* Values are expressed as means ± standard deviation (SD). Values followed by the same letter within a column are not significantly different (*p* ≤ 0.05).

**Table 2:** Effects of PVS2 dehydration duration on shoot regeneration from non-cryopreserved (−LN) and cryopreserved (+LN) shoot apices.

*Rosa* Genotypes		Shoot Regeneration (% ± SD) *							
		PVS2 Dehydration Duration (min)							
		0	10	15	20	25	30	35	40
‘Ioana’	−LN	96.6 ± 0.5 ^a^	88.3 ± 1.6 ^a^	80.0 ± 1.5 ^a,b^	75.0 ± 1.3 ^a,b^	75.0 ± 1.8 ^a,b^	71.6 ± 2.4 ^a,b^	55.0 ± 3.5 ^b,c^	33.3 ± 2.1 ^c^
	+LN	0 c	8.30 ± 1.3 ^b,c^	21.6 ± 1.8 ^b^	45.0 ± 1.5 ^a^	56.6 ± 1.2 ^a^	65.0 ± 1.8 ^a^	46.6 ± 2.2 ^a^	18.3 ± 1.9 ^b,c^
‘Mariana’	−LN	88.3 ± 0.7 ^a^	85.0 ± 1.3 ^a^	78.3 ± 1.1 ^a,b^	75.0 ± 1.8 ^a,b^	70.0 ± 1.6 ^a,b^	68.3 ± 2.0 ^a,b^	56.6 ± 3.0 ^b^	25.0 ± 1.3 ^c^
	+LN	0 ^d^	0 ^d^	26.6 ± 1.6 ^b,c^	46.6 ± 1.9 ^a,b^	51.6 ± 1.8 ^a^	58.3 ± 2.5 ^a^	50.0 ± 2.6 ^a^	15.0 ± 1.7 ^c,d^
‘Vulcan’	−LN	91.6 ± 0.9 ^a^	80.0 ± 1.4 ^a^	78.3 ± 1.4 ^a^	73.3 ± 1.6 ^a,b^	71.6 ± 2.1 ^a,b^	73.3 ± 1.7 ^a,b^	55.0 ± 1.8 ^b^	31.6 ± 1.9 ^b^
	+LN	0 ^e^	11.3 ± 1.8 ^d,e^	26.6 ± 1.5 ^c,d^	41.6 ± 2.1 ^b,c^	56.6 ± 2.2 ^a,b^	71.7 ± 2.8 ^a^	53.3 ± 2.1 ^a,b^	21.6 ± 1.7 ^c,d,e^

* Values are expressed as means ± standard deviation (SD). Shoot tips were osmoprotected in 0.75 M sucrose for 24 h. Values followed by the same letter within a row are not significantly different (*p* ≤ 0.05).

**Table 3:** Effects of liquid nitrogen storage on the multiplication index and the height of shoots from non-cryopreserved (−LN) and cryopreserved (+LN) shoot tips.

*Rosa* Cultivars	Cryopreservation Procedure	Response of Explants			
		Multiplication Index (nr. ± SD) *		Height of Shoots (cm ± SD)	
		−LN	+LN	−LN	+LN
‘Ioana’	DV	5.5 ± 1.3 ^a^	5.1 ± 1.1 ^a,b^	5.3 ± 1.8 ^a^	5.0 ± 1.6 ^a^
	ED	4.8 ± 1.7 ^a^	4.3 ± 1.0 ^b^	4.8 ± 1.5 ^a^	4.8 ± 1.0 ^a^
‘Mariana’	DV	5.8 ± 1.4 ^a^	5.5 ± 1.6 ^a,b^	4.3 ± 1.6 ^a^	4.9 ± 1.4 ^a^
	ED	5.1 ± 1.7 ^a^	4.6 ± 1.9 ^a,b^	4.8 ± 1.4 ^a^	4.7 ± 1.7 ^a^
‘Vulcan’	DV	6.5 ± 1.0 ^a^	6.3 ± 1.0 ^a^	6.0 ± 1.0 ^a^	5.8 ± 1.3 ^a^
	ED	5.8 ± 0.7 ^a^	5.3 ± 1.5 ^a,b^	4.7 ± 1.4 ^a^	5.0 ± 1.1 ^a^

* Values represent means ± standard deviation (SD). Non-cryopreserved shoot tips in the DV procedure were osmoprotected in sucrose (0.75 M for both procedures) and dehydrated for 30 min in PVS2; in the ED, the encapsulated shoot tips were osmoprotected in sucrose, and the moisture content of alginate beads was 22% cv. ‘Ioana’, 19% cv. ‘Mariana’ and 21% cv. ‘Vulcan’ after 4 h desiccation in laminar air flow. Values followed by different letters within a column indicate significant differences (*p* ≤ 0.05); DV: droplet-vitrification, ED: encapsulation-dehydration.

**Table 4:** Chlorophyll and carotenoid content in leaves derived from non-cryopreserved (−LN) and cryopreserved (+LN) shoot tips after droplet-vitrification and encapsulation-dehydration.

*Rosa* Cultivars	Procedure	Chlorophyll *a* (mg/g FW ± SD) *	Chlorophyll *b* (mg/g FW ± SD)	Carotenoids (mg/g FW ± SD)
	Droplet-vitrification			
‘Ioana’	−LN	0.91 ± 0.10 ^a^	0.51 ± 0.11 ^a^	0.04 ± 0.00 ^b^
	+LN	0.85 ± 0.09 ^a^	0.48 ± 0.07 ^a^	0.04 ± 0.00 ^b^
‘Mariana’	−LN	0.85 ± 0.13 ^a^	0.34 ± 0.10 ^a^	0.08 ± 0.00 ^a^
	+LN	0.88 ± 0.11 ^a^	0.27 ± 0.06 ^a^	0.08 ± 0.00 ^a^
‘Vulcan’	−LN	0.76 ± 0.02 ^a^	0.44 ± 0.07 ^a^	0.05 ± 0.00 ^b^
	+LN	0.74 ± 0.01 ^a^	0.46 ± 0.11 ^a^	0.05 ± 0.00 ^b^
	Encapsulation-dehydration			
‘Ioana’	−LN	0.93 ± 0.09 ^a^	0.55 ± 0.12 ^a^	0.04 ± 0.00 ^b^
	+LN	0.88 ± 0.07 ^a^	0.49 ± 0.03 ^a^	0.04 ± 0.00 ^b^
‘Mariana’	−LN	0.86 ± 0.14 ^a^	0.37 ± 0.03 ^a^	0.08 ± 0.00 ^a^
	+LN	0.90 ± 0.10 ^a^	0.31 ± 0.16 ^a^	0.09 ± 0.00 ^a^
‘Vulcan’	−LN	0.72 ± 0.03 ^a^	0.43 ± 0.11 ^a^	0.06 ± 0.00 ^b^
	+LN	0.74 ± 0.04 ^a^	0.47 ± 0.08 ^a^	0.05 ± 0.00 ^b^

* Values represent means ± standard deviation (SD). Values followed by the same letter within a column are not significantly different (*p* ≤ 0.05).

## Data Availability

The data presented in this study are available on request from the first author.

## Supplementary Materials

The following are available online at https://www.mdpi.com/article/10.3390/plants11081095/s1, Table S1. Morphological and horticultural characteristics of rose genotypes used in cryopreservation studies.
