# Supplementary material for: Cryo-Technologies for Ex Situ Conservation of Rosa Germplasm
Source: Plants (Basel). 2022 Apr 18;11(8):1095. doi: 10.3390/plants11081095 (PMC9027578; doi:10.3390/plants11081095)
Supplement: Supplementary file 1 [file plants-11-01095-s001.zip › plants-1660387-supplementary.pdf]

Table S1. Morphological and horticultural characteristics of rose genotypes used in cryopreservation studies.

Character description	*Rosa* x *hybrida* (L.) genotypes		
	´Ioana´	´Mariana´	´Vulcan´
	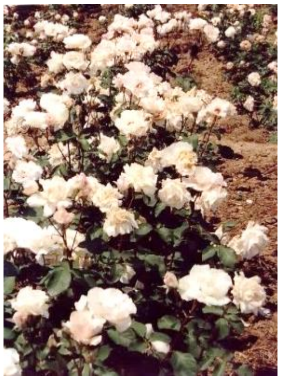	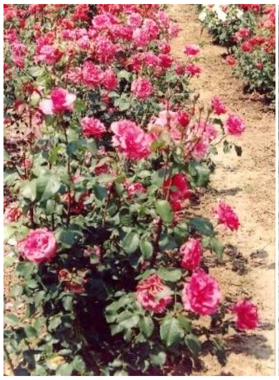	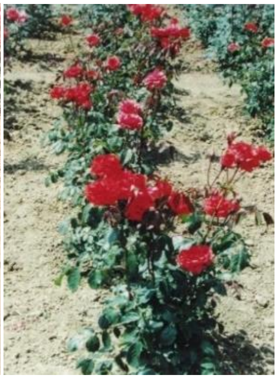
Plant habitus	upright	upright	upright
Plant height (cm)	91 ± 1.52a*	90 ± 1.55a	79.6 ± 1.62b
Young shoot (20 cm in length)	strong	strong	medium
Leaf length/color	medium/medium	high/intense	high/intense
Leaflet: undulation of margin	weak	strong	strong
Leaflet transversal section	slightly concave	slightly concave	straight
Glossiness of the upper surface of the leaf	weak	strong	strong
	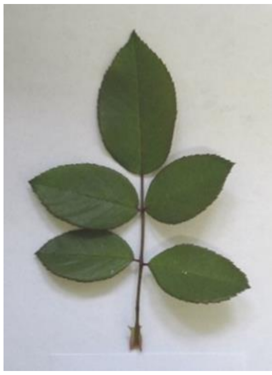	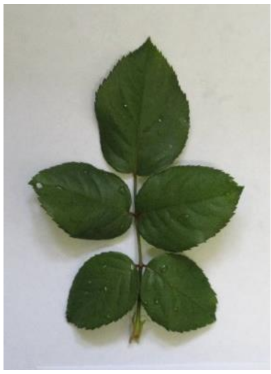	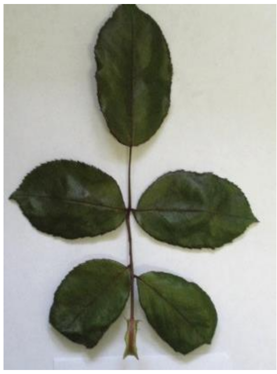
Thorn shape at top/base	curved/curved	curved/curved	curved/curved
	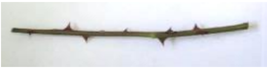	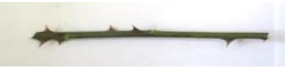	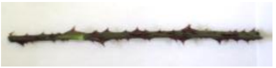
Number of short thorns (length<5 mm)	small	small	very high
Number of long thorns (length>5 mm)	medium	medium	medium
Flowering shoots (number of flowers)	medium (5)	medium (5)	very high (9)

	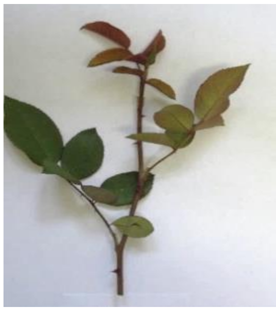	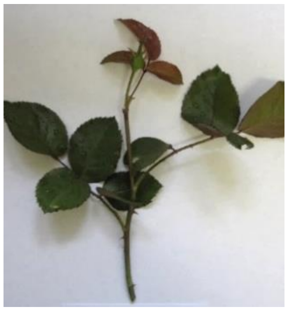	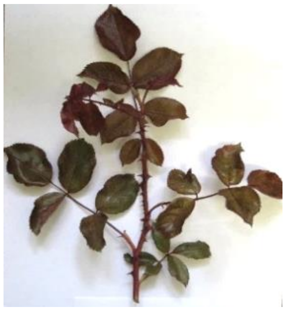
Flowering	early blooming; flowering period 104 days	early blooming; flowering period 96 days	late blooming; flowering period 87 days
Flower peduncle (number of hairs or thorns)	small	small	medium
	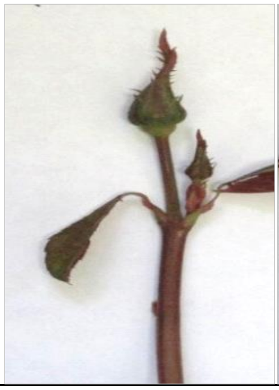	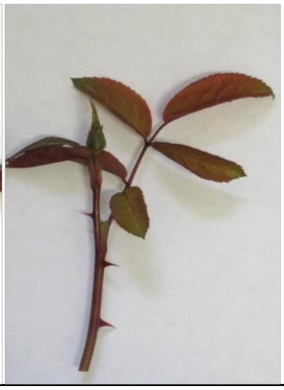	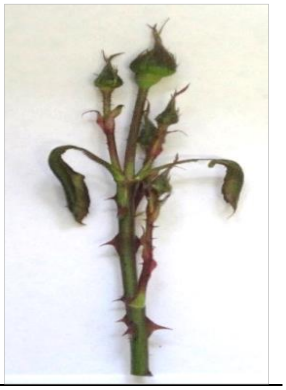
Floral bud	ovoid	ovoid	globose
	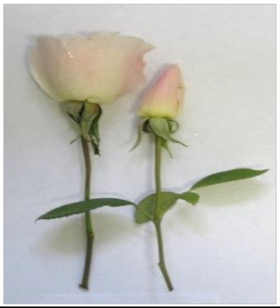	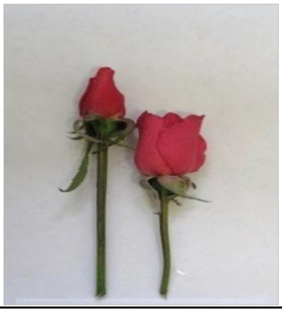	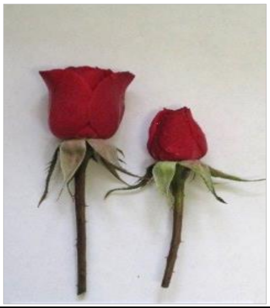
Inflorescence type	semi-double	double	double
	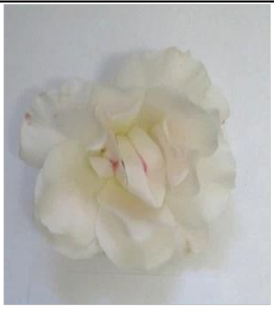	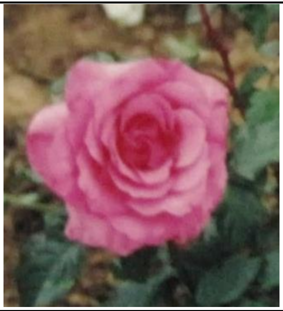	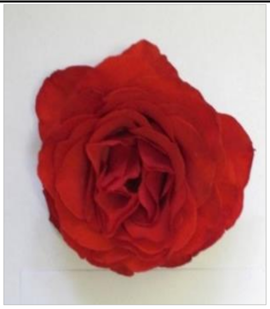
Corolla shape	irregularly rounded	irregularly rounded	irregularly rounded
Flower color	white	pink	red
Diameter of flowers (cm)	10.3 ± 1.74a	11.6 ± 1.52a	7.8 ± 1.83b
Number of petals/flower	20 ± 1.55b	35 ± 1.73a	34.4 ± 2.15a

Petals size/shape	medium/obovate	high/obovate	medium/round
Sepals	short	medium	medium
Stamen (predominant color of filament)	pink	pink	yellow
Style (size/color)	medium/green-yellow	medium/green-yellow	medium/green-yellow

*Values represent means ± standard deviation (SD); Values followed by the same letter within a row are not significantly different ($P \leq 0.05$).
